# Patient experience: Feeling like the tin man from the Wizard of Oz

**DOI:** 10.1002/ski2.406

**Published:** 2024-06-14

**Authors:** Caoimhe Dalton, Lisa Murphy, Carmel Ann Galligan, Susan O’Gorman, Larry Bacon, Claudine Howard‐James, Rachel Dillon, Holly Fitzgerald

**Affiliations:** ^1^ St James's Hospital Dublin Ireland

## Abstract

Graft versus host disease affects roughly 40%–60% of patients who undergo haematopoietic stem cell transplant, with a mortality rate of 15%. In rare cases, this can progress to sclerodermatous graft versus host disease, with devastating associated morbidity and mortality. In this article, a patient shares their first‐hand experience of living with the disease.

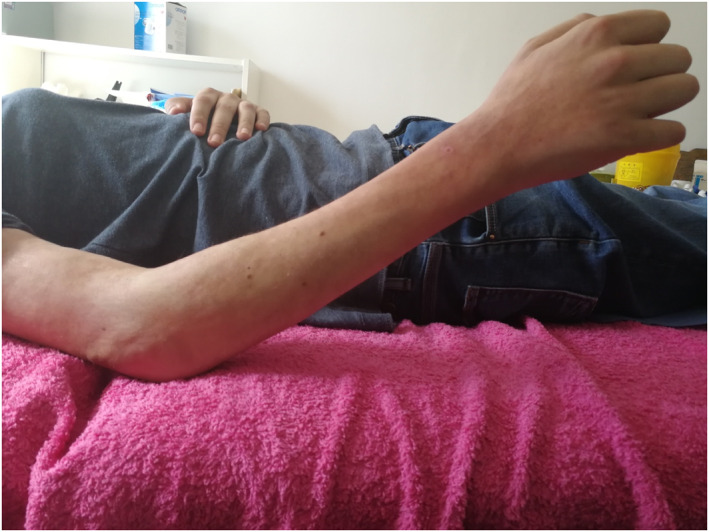

## 1 PATIENT’S PERSPECTIVE

In 2014, aged 17, I got what I thought was a cold that didn't go away. Before that, I was relatively active with a normal teenage life. I went for blood tests, but they never really spotted anything. I had a chest x‐ray done and 30 min after, a nurse came in and said, ‘you're very sick’. I was brought to the hospital in an ambulance thinking I had a pneumonia. Following a bone marrow aspirate, I was given a diagnosis of acute lymphoblastic leukaemia. I met the consultant, and she said that I would be there for a couple of months of chemotherapy. I was there for over 15 months and I only got home for a couple of weeks at a time.

I had the option for a transplant a couple of months before I went for it. I was scared by the risks of not having an immune system and wondering how the new immune system would behave. After multiple courses of failed chemotherapy, I underwent transplant from a matched unrelated donor on the 29th of April 2016.

**FIGURE 1:**
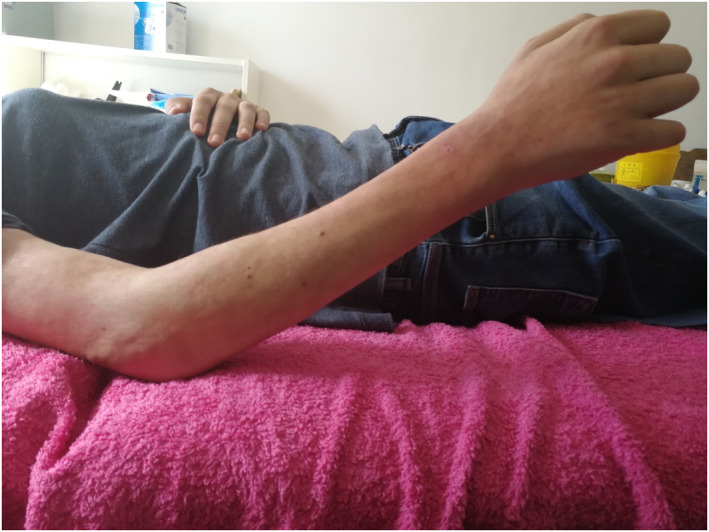
Patient's right arm fixed in flexion at the elbow joint with evidence of skin tightening and reduced skin wrinkling at the metacarpophalangeal joints.

**FIGURE 2:**
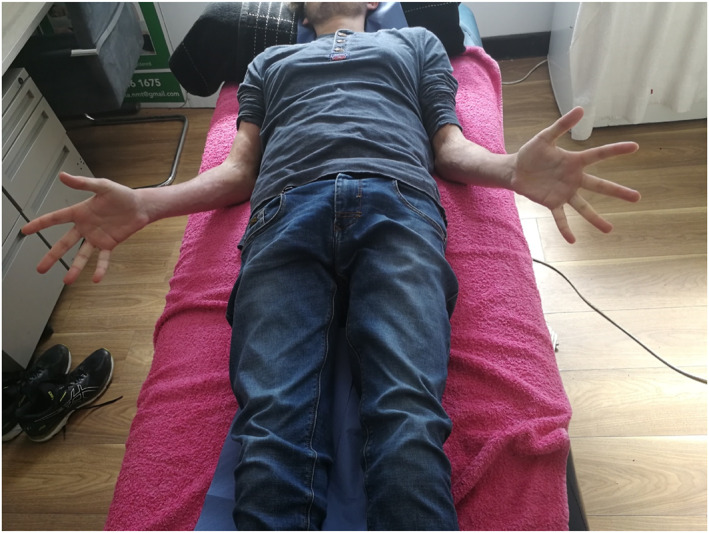
Patient's arms held in flexion at the elbow joints bilaterally with evidence of shiny, tightened skin and pallor of the third‐fifth digits.

As far as I remember, for about a year post‐transplant, everything was perfect. The doctors were kind of shocked nothing was going wrong. On my 20th birthday, I was going for a walk along the pier in Wicklow and I said to my dad, ‘’I don't think I can stretch out my arms as much as I used to’’. Shortly after, this had progressed to involving my wrists, ankles, and shoulders (Figure 1). I felt like the tin man from the Wizard of Oz with my arms stuck at 90‐degree angles (Figure 2). My skin started getting very weak and I developed lots of sores and blisters. It also became tight and tough like leather. No matter how hard I tried, how I sat down or twisted or bent, I couldn't pick up anything off the floor. Putting on a pair of socks was impossible. I mentioned it to my consultant and a while after that I started photopheresis. The specialist nurse examined my skin and told me what would be involved with therapy. I was reassured by everyone that it would be painless, as I was worried it was going to be another form of chemotherapy.

By the time I was going for photopheresis, everywhere in my body was affected except for my face. I had seen a dermatologist who prescribed a bunch of different ointments and creams. They gave me zip socks to wear on my legs and arms to try to stop the blisters from forming but the blisters would form under the socks, fusing them into my skin. The occupational therapist made me custom splints that I wore to hold my arms and wrists at the widest angle that I could manage to slowly stretch the skin over months.

I was taking tablets (imatinib and mycophenolate mofetil) for a while before the photopheresis. Day to day, after 4 years, I still don't really notice a difference, but if I look back, the change is obvious. In fact, it was a massage therapist that was regularly applying oils to my skin who first noticed improvement. After that, I started realizing the blisters were gone and the skin in certain parts was beginning to get a bit looser.

I don't do a whole lot for my skin anymore. For about a year, I was using all the prescribed creams, ointments, hair, and body washes, but these were expensive, and I got tyred from constantly applying them. My partner still puts cream on my back every night and I would have to say that my back is in the best condition out of all my affected skin. It seems obvious and I know for a fact it would help so it is something I've been trying to do a bit more.

It's been a very slow process. My earlier attitude towards everything medical was that things would usually sort themselves out. Unfortunately, that's not exactly how graft versus host disease (GVHD) of the skin behaves, and I know that I need to do my part with continuous therapy.

## 2 CLINICAL SUMMARY

GVHD affects roughly 40%–60% of patients who undergo haematopoietic stem cell transplant, with a mortality rate of 15%.^1^ GVHD is a CD‐8 T cell mediated process whereby donor cells recognise host major histocompatibility complex proteins in tissues as foreign material. This results in a type IV cytotoxic T cell hypersensitivity reaction between the donor and the recipient. The classification of acute and chronic GVHD was redefined in 2005 at the National Institutes of Health (NIH) Consensus Conference and divided into two subcategories (classic acute GVHD and persistent, recurrent or late‐onset acute GVHD; classic chronic GVHD and overlap syndrome). The subcategories were based predominantly on clinical and histopathological features rather than timing post‐transplant alone. This framework was reviewed by the NIH in 2014 and maintained with further diagnostic refinement.^2^, ^3^

Inamoto et al. reported that the incidence of sclerotic GVHD (ScGVHD) is 20% by 3 years after initial treatment for chronic GVHD.^4^ ScGVHD has varying degrees of cutaneous, mucocutaneous, and internal organ involvement. It typically presents with a combination of atrophy, sclerosis, telangiectasias, hyperpigmentation, contractures, alopecia, ulcerations and koilonychia.^5^

Management with psoralen plus ultraviolet A, narrowband ultraviolet B therapy and extracorporeal photopheresis have had varying success in the event of failure of conventional immunosuppressant therapy.^6^ Investigation into novel treatments including tyrosine kinase inhibitors and selective JAK inhibitors is ongoing.^7^, ^8^ Often several treatment modalities are employed, as was the case with our patient. Appropriate patient education and multidisciplinary input are critical aspects of management.

## CONFLICT OF INTEREST STATEMENT

The authors declare no conflicts of interest.

## AUTHOR CONTRIBUTIONS

**Caoimhe Dalton**: Conceptualization (equal); investigation (equal); writing – original draft (equal); writing – review & editing (equal). **Lisa Murphy**: Conceptualization (equal); writing – review & editing (equal). **Carmel Ann Galligan**: Conceptualization (equal); visualization (equal); writing – review & editing (equal). **Susan O’Gorman**: Conceptualization (equal); supervision (equal). **Larry Bacon**: Conceptualization (equal); visualization (equal). **Claudine Howard‐James**: Writing – review & editing (equal). **Rachel Dillon**: Writing – review & editing (equal). **Holly Fitzgerald**: Writing – review & editing (equal).

## ETHICS STATEMENT

Not applicable.

## PATIENT CONSENT

Written patient consent for publication was obtained.

## Data Availability

Data sharing is not applicable to this article as no new data were created or analyzed in this manuscript.
